# Physicochemical, Nutritional Properties and Metabolomics Analysis Fat Deposition Mechanism of Chahua Chicken No. 2 and Yao Chicken

**DOI:** 10.3390/genes13081358

**Published:** 2022-07-28

**Authors:** Yong Liu, Shuangmin Liang, Kun Wang, Xiannian Zi, Ru Zhang, Guangzheng Wang, Jiajia Kang, Zijian Li, Tengfei Dou, Changrong Ge

**Affiliations:** 1 College of Animal Science and Technology, Yunnan Agricultural University, Kunming 650201, China; lydzq05091025@163.com (Y.L.); wangkun@126.com (K.W.); zxnzyx1119@163.com (X.Z.); ynauliuyong@126.com (R.Z.); w18887643417@163.com (G.W.); kjj730248@163.com (J.K.); lzj01071217@163.com (Z.L.); tengfeidou@sina.com (T.D.); 2 College of Food Science and Technology, Yunnan Agricultural University, Kunming 650201, China; liangsm061211@126.com

**Keywords:** chicken, meat quality characteristics, fat deposition, metabonomics

## Abstract

Poultry is an important dietary source of animal protein, accounting for approximately 30% of global meat consumption. Because of its low price, low fat and cholesterol content, and no religious restrictions, chicken is considered a widely available healthy meat. Chahua chicken No. 2 is a synthetic breed of Chahua chicken derived from five generations of specialized strain breeding. In this study, Chahua chicken No. 2 (CH) and Yao chicken (Y) were used as the research objects to compare the differences in physicochemical and nutritional indicators of meat quality between the two chicken breeds, and metabolomics was used to analyze the differences in metabolites and lipid metabolism pathways and to explore the expression of genes involved in adipogenesis. The physical index and nutritional value of CH are better than that of Y, and the chemical index of Y is better than that of CH. However, the chemical index results of CH are also within the normal theoretical value range. Comprehensive comparison shows that the meat quality of CH is relatively good. Metabolomics analysis showed that CH and Y had 85 different metabolites, and the differential metabolites were mainly classified into eight categories. KEGG pathway enrichment analysis revealed 13 different metabolic pathways. The screened *PPARG*, *FABP3*, *ACSL5*, *FASN*, *UCP3* and *SC5D* were negatively correlated with muscle fat deposition, while *PPARα*, *ACACA* and *ACOX1* were positively correlated with muscle fat deposition. The meat quality of CH was better than Y. The metabolites and metabolic pathways obtained by metabonomics analysis mainly involved the metabolism of amino acids and fatty acids, which were consistent with the differences in meat quality between the two breeds and the contents of precursors affecting flavor. The screened genes were associated with fatty deposition in poultry.

## 1. Introduction

With the rapid development of the livestock and poultry breeding industry and the continuous improvement of people’s living standards, the demand for meat, eggs and milk is also increasing, and consumers’ demand for meat products has also changed from the pursuit of output to the requirements of quality [1]. Chicken accounts for a high proportion of livestock and poultry meat products [2], and has become one of the most popular meat products in the world due to its unique flavor, rich nutrition, high protein and low fat. The quality of chicken has a significant impact on the meat market and dominates consumers’ choice and purchase demand for chicken [3,4]. Therefore, cultivating high-quality chicken is a key concern.

The Chahua chicken is a native breed of southwestern China and is considered to be a primitive type of chicken that exhibits many phenotypes and behaviors similar to those of the red jungle chicken. The adult weight is about 1190 g for males and about 1000 g for females [5,6,7]. CH is based on Chahua chicken, which is one of the “six famous chickens in Yunnan Province”, and is a matching line variety that has been selected and bred through five generations of specialized lines. The cultivated CH commercial generation is 120 days old, and the slaughter weight is 2250–2350 g for males and 1850–1950 g for females, which not only retains the variety advantages of the original breed, but also overcomes the shortcoming of its small size and has high economic value. Y is an indigenous chicken breed from Guangxi, China, and it is known for its low fat deposition and better meat quality, but low egg production [8]. The commercial generation of Y is 120 days old, and the slaughter weight is 2480–2600 g for males and 1750–1830 g for females. In the current domestic chicken consumption market, it is favored by consumers, and the market prospect is very good. There are also obvious differences in the meat quality of chickens due to different types of chickens, age, genetic factors and their metabolic differences [9]. Meat quality is a complex comprehensive trait, with comprehensive feelings reflected in consumers, including sensory evaluation, physical indicators, chemical indicators, histological indicators, nutritional value, etc. At present, the nutritional value of chicken has received attention, but the physical and chemical properties of meat, including meat color [10], pH [9], dehydration rate [11], tenderness [12], etc., are also important factors in meat processing and consumer acceptance.

In the poultry development industry, while pursuing conditions such as chicken growth rate, egg production rate and adapting to market demand, it is also necessary to pay attention to the increased fat deposition in chicken, which will affect the meat quality and health of chicken [13]. Intramuscular fat content is an important indicator of poultry meat quality and an important economic trait. At present, the progress of intramuscular fat breeding by conventional breeding methods is slow. Therefore, in order to improve the selection efficiency and speed up the genetic progress, molecular genetic markers can be used for assisted selection. In the wave of omics development, more and more people use omics sequencing technology to mine candidate genes that affect chicken quality, providing theoretical support for molecular marker-assisted breeding [14]. Metabolomics seeks the relative relationship between metabolites and physiological or pathological changes by quantitatively analyzing metabolites in organisms [15,16,17]. Compared with genomics, metabolomics can amplify small changes in gene and protein expression levels at the level of metabolites, and can instantly and sensitively reflect changes in the functional state of organisms caused by internal and external factors such as genes or the environment. Finally, it helps us understand the metabolism law of livestock and poultry at the molecular level [18]. Wen et al. [19] used metabolomics to measure the quality of refrigerated chicken and found that changes in refrigeration time caused most of the amino acid changes. Yuan et al. [20] used metabolomic analysis to find that cellobiose, mannose and allose in chicken duodenum may be related to the co-degradation of lignocellulose by *Rhodococcus*.

Therefore, the purpose of this experiment was to study the differences in meat physicochemical and nutritional indicators between CH and Y, to analyze the differences in metabolites and lipid metabolism pathways between the two chicken species by metabolomics, and to explore the gene expression of the mechanism of fat formation.

## 2. Materials and Methods

### 2.1. Animal Experimentation Ethical Statement

All studies involving animals were conducted in accordance with the Regulations on the Administration of Laboratory Animal Affairs (Ministry of Science and Technology of China; revised June 2004). All procedures conducted with the chickens were approved by the Yunnan Agricultural University Animal Care and Use Committee (approval ID: YAU ACU C01). Sample collection was performed in accordance with the “Guide for the Care and Use of Laboratory Animals of Yunnan Agricultural University” and the “Guide for the Care and Use of Laboratory Animals” published by the England National Research Council.

### 2.2. Chicken, Diet and Housing

This study was carried out at Yunnan Agricultural University (Kunming, China). CH and Y eggs were incubated in the teaching practice chicken farm of Yunnan Agricultural University. A total of 200 chickens of 1 day of age (100 from each chicken breed) were reared under standard conditions on a starter diet (Period I: 20.0% CP and 13.02 MJ/kg ME) to 30 days of age. From 30 days of age onward, chickens were fed a regular diet (Period II: 18.6% CP and 12.8 MJ/kg ME) to 120 days. Diet content was consistent with the formulation to meet NRC 1994 and Chinese Chicken Feeding Standard recommendations [21,22,23]. The compositions of diet details are shown in Table S1 of the Supplementary Materials. Immunization was carried out according to routine immunization procedures. Both males and females were raised in the same pen with the density of 20 birds/m^2^ of floor area from 0 days to 28 days and 12 birds/m^2^ of floor area from 29 days to 56 days. From 59 days to 120 days, males and females were raised separately with the density of 5 birds/m^2^ of floor area. All chickens were sacrificed at 120 days.

### 2.3. Slaughter Procedure and Sample Collecting

Feed was withdrawn 16 h and water 12 h before slaughter. The body weight of the chickens was measured at 120 days of age in the morning. CH and Y 60 each (half male and half female) were selected. Chickens were slaughtered by cervical dislocation in accordance with the National Experimental Animal Slaughter Standard of China. Left pectoral muscle and leg muscle tissues (100 mg) were quickly collected in 2 mL sterile tubes (RNase-free) and frozen in liquid nitrogen, and then transferred to a −80 °C ultra-low-temperature refrigerator for subsequent analysis of gene expression in qRT-PCR. The remaining left pectoral muscle and leg muscle were removed from the fascia and stored in a Ziplock bag in a refrigerator at −20 °C for future use for the determination of muscle quality physical and chemical indexes, amino acid content, fatty acid and metabolomics.

### 2.4. Determination of Muscle Physical Parameters

Muscle color was measured by a color difference meter (Suzhou Yuhong Trading Co. LTD, Suzhou, China), muscle pH was measured by a portable pH meter (Hanna-HI9025, Milan, Italy), muscle shear force was measured by a strain-controlled unconfined pressure meter (Nanjing Soil Instrument Factory Co. LTD, Nanjing, China) and water loss rate was calculated by the following formula: water loss rate (%) = (W1 − W2)/W1 × 100%. (W1: weight before pressing; W2: weight after pressing).

### 2.5. Determination of Routine Muscle Chemical Parameters

The fresh muscle samples were pounded and placed in a freeze-drying machine (FD-1C-50, Beijing Boyikang Test Instrument Co. LTD, Beijing, China) to a constant weight, then crushed with a multi-functional grinder (DC-1000A, Zhejiang Wuyi Dingzang Daily Metal Products Factory, Jinhua, China) for later use. Water content, CP content and ash content were determined in strict accordance with Chinese national standards.

### 2.6. Determination of Amino Acid Content

Amino acid content was determined by an automatic amino acid analyzer (S433D, Sykam, Munich, Germany). The contents of proline and other amino acids were determined at 440 nm and 570 nm, respectively.

### 2.7. Determination of Fatty Acids

Fatty acids were determined by gas mass spectrometry (Trace 1310-ISQ 7000, Thermo Fisher, Waltham, MA, USA). The injection volume was 1 μL, the split ratio was 8:1, the inlet temperature was 250 °C, the ion source temperature was 230 °C, the transmission line temperature was 250 °C, the quadrupole temperature was 150 °C and the carrier gas flow rate was 0.63 mL/min.

### 2.8. Metabolomics Determination

#### 2.8.1. LC-MS Analysis for Metabolome Analysis

LC-MS was detected by liquid chromatography (UltiMate 3000, Thermo, Waltham, MA, USA) and mass spectrometry (Q Exactive Focus, Thermo, Waltham, MA, USA). The injector temperature was set at 8 °C, the flow rate was 0.25 mL/min, the column temperature was set at 40 °C, and the positive ion mobile phase was 0.1% formic acid water and −0.1% formic acid acetonitrile (anion 5 mM ammonium formate water (A), acetonitrile (B)).

#### 2.8.2. Identification of Differential Metabolites

Differential metabolites were searched by screening metabolites. Detailed parameters of screening related differential metabolites are shown in Table S2 of the Supplementary Materials.

#### 2.8.3. Differential Metabolite Heat Map Analysis and Metabolite Pathway Analysis

The hierarchical cluster diagram of relative quantitative values of metabolites was obtained by the PheatMap program package (PNMK-4-KYTD012-A/0, Jiangsu, China), with columns representing samples and rows representing metabolites. The relative value of metabolites under different test conditions was the metabolic level. KEGG metabolic pathways were enriched, and possible metabolic pathways were identified, and then metabolic pathways of metabolites were analyzed.

### 2.9. qRT-PCR Verification

Nine candidate genes were selected for qRT-PCR validation. After extracting total RNA from breast muscle and leg muscle tissue, the total RNA reverse transcriptase kit (Takara, Kusatsu, China) was used to synthesize cDNA. Real-time PCR ABI 7500 Fast Real-Time PCR System using SYBR premix Ex TaqTM II (Takara, Kusatsu, China) was used to perform real-time PCR. The 2^−ΔΔCT^ method was used to determine relative expression, and β-actin was used as the internal control for normalization of the results. All primer sequences were listed shown in Table S3 of the Supplementary Materials.

### 2.10. Statistical Analysis

Excel 2013 (Microsoft Office, Washington, DC, USA) was used to process data, SPSS 21.0 (SPSS, Chicago, IL, USA) was used for statistical analysis, and the results were expressed as (mean ± standard deviation); Duncan’s method was used for significant difference analysis, and Analyze–Correlate–Bivariate was used for correlation analysis. Quality control QC and QA were performed on the exported data through Proteowizard software (v3.0.8789, Jiangsu, China), XCMS package of R (v3.3.2, Auckland, New Zealand), etc., to obtain reliable and high-quality metabolomics data. Relative quantitative PCR was used to detect the amount of target gene and internal reference gene. The relative expression of genes is obtained by processing the data and calculating by Excel 2013. The calculation method is as follows: Ratio = 2^−∆CT(CH-β-actin)^/2^−∆CT(Y-β-actin)^.

## 3. Results

### 3.1. Comparative Body Weight Analysis

**Figure 1:**
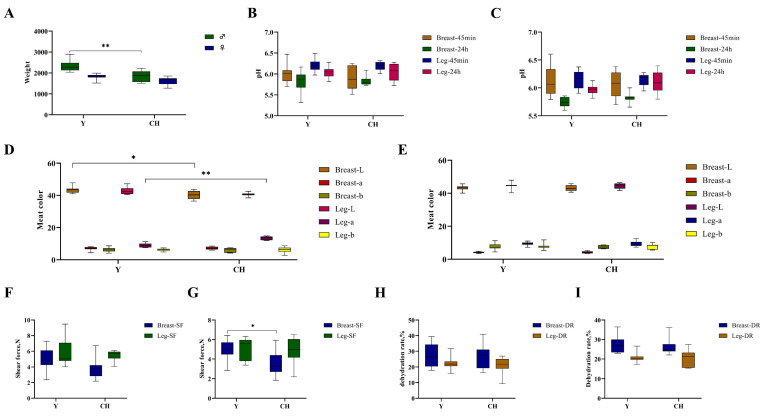
Comparison of body weight and meat quality physical indexes between Y and CH. (**A**) Comparison of body weight of two chicken breeds. (**B**) Comparison of pH values between two males. (**C**) Comparison of pH values between two females. (**D**) Comparison of meat color between two males. (**E**) Comparison of meat color between two females. (**F**) Comparison of shear force between two males. (**G**) Comparison of shear force between two females. (**H**) Comparison of dehydration rate between two males. (**I**) Comparison of dehydration rate between two females. ** indicates extremely significant differences (*p* < 0.01); * indicates significant difference (*p* < 0.05).

The comparison of body weight between males and females is shown in Figure 1A. The male and female body weights of Y were higher than those of CH. Y males and females weighed 2333.60 ± 150.52 g and 1824.00 ± 78.74 g, respectively. CH males and females weighed 1844.55 ± 102.42 g and 1611.05 ± 48.37 g, respectively. The male body weight of Y was significantly higher than that of CH (*p* < 0.01).

### 3.2. Comparative Analysis of Physical Indexes

#### 3.2.1. pH

The comparison of pH values between Y and CH males is shown in Figure 1B. The pH of the breast and leg muscles of Y was higher than that of CH at 45 min, and the pH of breast and leg muscles of CH was higher than that of Y at 24 h. The observed pH 24 h and pH 45 min values in the breast and leg muscles of Y and CH were within the normal range (5.6–6.4) and there was no difference.

The comparison of pH values between Y and CH females is shown in Figure 1C. The pH of the breast and leg muscles of Y was higher than that of CH at 45 min, and the pH of breast and leg muscles of CH was higher than that of Y at 24 h. The observed pH 24 h and pH 45 min values in the breast and leg muscles of Y and CH were within the normal range (5.6–6.4), and the difference was not significant.

#### 3.2.2. Meat Color

The comparison of meat color (L, a and b values) between Y and CH males is shown in Figure 1D. The L and b values of the breast and leg muscles of Y were higher than those of CH, and the L value of the breast muscle of Y was significantly higher than that of CH (*p* < 0.05). The a values of the breast and leg muscles of CH were higher than those of Y, and the a values of the leg muscles of CH were significantly higher than those of Y (*p* < 0.01). (Breast muscles (Y: L 43.40, a 6.98, b 6.19. CH: L 40.32, a 7.18, b 6.09); leg muscles (Y: L 42.82, a 8.91, b 6.21. CH: L 40.59, a 13.46, b 6.12).)

The comparison of meat color (L, a and b values) between Y and CH females is shown in Figure 1E. The L and b values of the breast and leg muscles of Y were higher than those of CH. The a values of the breast and leg muscles of CH were higher than those of Y, and the differences were not significant (*p* > 0.05). (Breast muscles (Y: L 43.15, a 4.14, b 7.63. CH: L 43.08, a 4.18, b 7.43); leg muscles (Y: L 44.60, a 9.38, b 7.82. CH: L 44.53, a 9.44, b 7.81).)

#### 3.2.3. Shear Force

The comparison of shear force between Y and CH males is shown in Figure 1F. The shear force of breast muscle and leg muscle of Y was higher than that of CH, but the difference was not significant (*p* > 0.05). The observed shear force in the breast and leg muscles of Y and CH were within 3.76–5.47 N, and the difference was not significant.

The comparison of shear force between Y and CH females is shown in Figure 1G. The shear force of Y breast muscle and leg muscle was higher than that of CH, and the shear force of Y (4.93 N) breast muscle was significantly higher than that of CH (3.50 N) (*p* < 0.05).

#### 3.2.4. Dehydration Rate

The comparison of dehydration rate between Y and CH males is shown in Figure 1H. The dehydration rate observed in the breast muscles of Y and CH ranged from 26% to 27%, and in the leg muscles, from 20% to 22%. The dehydration rate of breast muscle and leg muscle of Y was higher than that of CH, but the difference was not significant (*p* > 0.05).

The comparison of dehydration rate between Y and CH females is shown in Figure 1I. The dehydration rate observed in the breast muscles of Y and CH ranged from 25% to 27%, and in the leg muscles, it was 20%. The dehydration rate of breast muscle and leg muscle of Y was higher than that of CH, but the difference was not significant (*p* > 0.05).

### 3.3. Comparative Analysis of Chemical Composition

The comparison of muscle chemical composition between Y and CH is shown in Table 1. The water, crude fiber (CF) and ash in muscle of Y were higher than those of CH. The crude protein (CP) in breast muscle and leg muscle of Y was significantly lower than in CH (*p* < 0.01).

### 3.4. Comparative Analysis of Amino Acid Content

The comparison of amino acids in muscles between Y and CH males is shown in Table 2. CH TAA content (*p* < 0.01), UAA content (*p* < 0.01) and EAA content (*p* < 0.05) were significantly higher than in Y; the contents of amino acids in muscles of CH were higher than in Y, except for Gly and Pro.

The comparison of amino acids in muscles between Y and CH females is shown in Table 3. The TAA and UAA contents in CH muscles were significantly higher than in Y (*p* < 0.01). The EAA and NEAA contents in CH muscles were higher than in Y, but the difference was not significant (*p* < 0.05). The contents of Gly and Pro in muscles of CH were lower than those of Y, and the contents of other amino acids were higher than Y.

### 3.5. Comparative Analysis of Fatty Acid Content in Muscle

The comparison of FA content in muscles between Y and CH males is shown in Table 4. The contents of TFA, EFA, PUFA and SFA in the breast and leg muscles of CH were lower than those of Y. The contents of PUFA and EFA in the breast muscle of CH were significantly lower than those of Y (*p* < 0.05), while the differences in the contents of SFA and TFA were not significant (*p* > 0.05). The contents of TFA, SFA, MUFA, PUFA and EFA in the leg muscle of CH were significantly lower than those of Y (*p* < 0.01). The content of fatty acids in the leg muscles and breast muscles of the two chickens was different. The fatty acid content of leg muscle and chest muscle is higher for C16:0, C18:0, C18:1N12, C18:1N9C, C18:2N6 and C20:4N6.

The comparison of FA content in muscle between Y and CH females is shown in Table 5. The contents of TFA, EFA, PUFA and MUFA in the breast and leg muscles of CH were lower than those of Y. The content of SFA in the breast muscle of CH was significantly lower than that of Y (*p* < 0.05), and the contents of TFA, EFA, PUFA and MUFA were significantly lower than those of Y (*p* < 0.01). The contents of TFA, EFA, MUFA and PUFA in the leg muscle of CH group were extremely significantly lower than in Y (*p* < 0.01), and SFA content was higher than in Y, but the difference was not significant (*p* > 0.05). The fatty acid content in the breast and leg muscles of the two chickens was SFA > PUFA > MUFA > EFA. The species with higher fatty acid content in the females was the same as for the males, but their contents were different.

### 3.6. Metabolomics Analysis

#### 3.6.1. Quality Control and Quality Assurance

**Figure 2:**
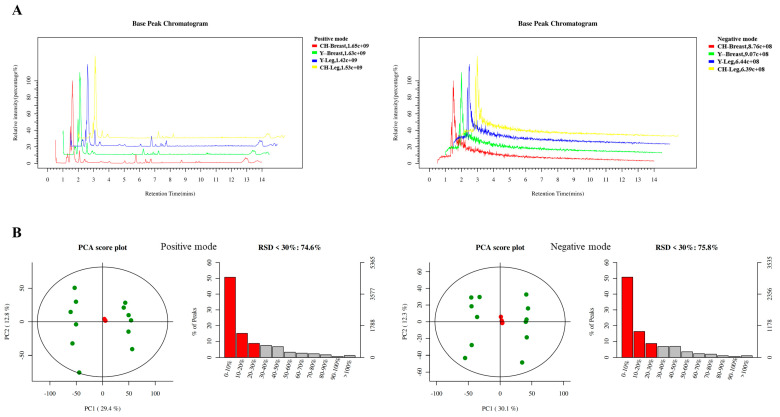
Metabolome quality control and quality assurance. (**A**) BPC diagram of positive and negative ion modes of samples. (**B**) PCA score chart of positive and negative ion mode of sample quality control.

There were differences between samples in positive and negative ion modes (Figure 2A). The sample reliability was within the 95% confidence interval, the QC samples were clustered with good repeatability and the system was stable. The characteristic peak ratios with RSD < 30% were up to 70%, and the data were satisfactory (Figure 2B).

#### 3.6.2. Principal Component Analysis and PLS-DA

**Figure 3:**
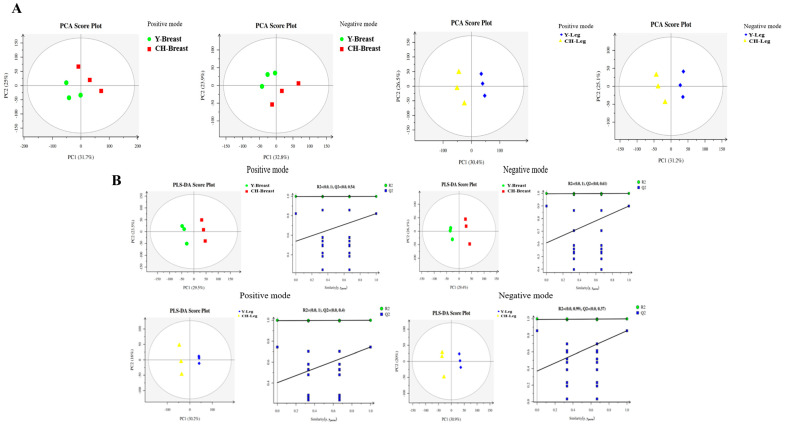
PCA score and PLS−DA. (**A**) PCA score of positive and negative ion mode in muscle tissue of two kinds of chickens. (**B**) PLS−DA score chart and replacement test chart of positive and negative ion mode in muscle tissue of two kinds of chickens.

The sample reliability was all within the 95% confidence interval, and the sample points of the same part of different chicken species were obviously separated, indicating that their metabolite profiles were different (Figure 3A). The breast muscle tissue and leg muscle tissue of the two chicken species were separated during principal component analysis, and the model was robust and reliable without any fitting (Figure 3B).

#### 3.6.3. Screening of Differential Metabolites

**Figure 4:**
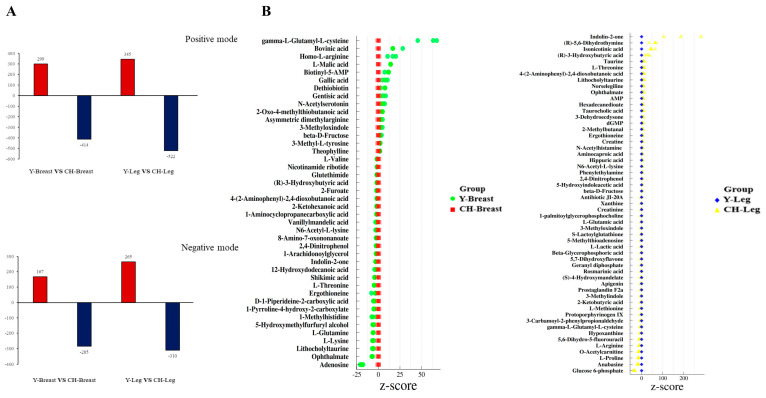
Screening and identification of differential metabolites. (**A**) Statistical results of different metabolites of breast muscle and leg muscle in positive and negative ion mode. (**B**) Z−score of different metabolites.

The statistical results of total differential metabolites in muscle tissue are shown in Figure 4A. The red metabolites were up-regulated and the blue metabolites were down-regulated. Comparison of metabolism in Y and CH muscles in positive ion mode: 299 metabolites in breast muscle were up-regulated, 414 metabolites were down-regulated; 345 metabolites in leg muscle were up-regulated, 522 metabolites were down-regulated. Comparison of metabolism in Y and CH muscles in negative ion mode: 167 metabolites were up-regulated and 285 metabolites were down-regulated in breast muscle; 265 metabolites were up-regulated and 310 metabolites were down-regulated in leg muscle.

#### 3.6.4. Identification of Differential Metabolites

The differential metabolites in the breast and leg muscles of Y and CH were identified. A total of 42 metabolites were identified in breast muscle tissues of Y and CH, including benzene-containing compounds, nucleoside and nucleotide acid analogues, organic acids and their derivatives, etc. (Table 6). A total of 55 metabolites were identified from leg muscle tissue, including benzene-containing compounds, organic acids and their derivatives, organic oxygenates, etc. (Table 7).

Standard scores of differential metabolites (Z-score) (Figure 4B) show that metabolites such as γ-L-glutamyl-L-cysteine, bovinic acid, homo-L-arginine and L-malic acid in breast muscle tissue had great differences between the two chicken breeds. The metabolites of indolin-2-one, (R)-5,6-dihydrothymine and isonicotinic acid in leg muscle tissue were significantly different between the two breeds.

#### 3.6.5. Cluster Analysis of Differential Metabolites

**Figure 5:**
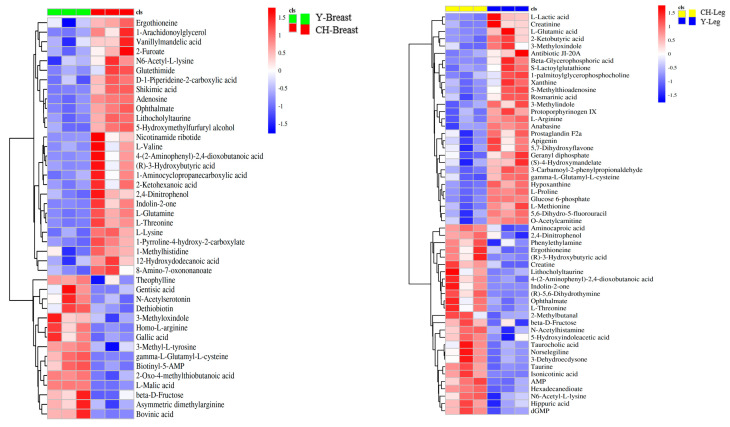
Thermogram of different metabolites in muscle tissue.

The hierarchical cluster diagram of relative quantitative value of metabolites was obtained through analysis, and the sizes of relative content are displayed by different colors, in which columns represent samples and rows represent metabolites. The heat map of metabolites in breast muscle and leg muscle tissue (Figure 5) shows that the expressions of differential metabolites were significantly differentiated between the two breeds and were relatively consistent with the expressions between breeds.

#### 3.6.6. Differential Metabolites KEGG Pathway and KEGG Pathway Enrichment

**Figure 6:**
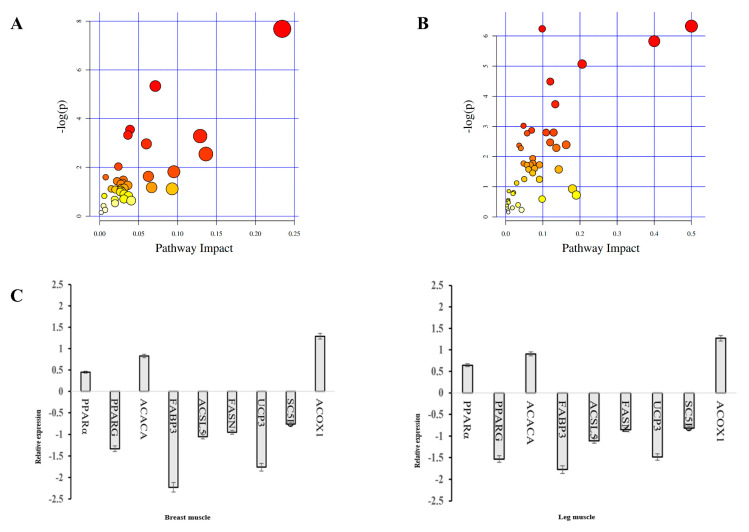
KEGG pathway enrichment. (**A**) Metabolic pathway influencing factors in breast muscle tissue. (**B**) Metabolic pathway influencing factors in leg muscle tissue. (**C**) Relative expression of gene.

Differential metabolites in breast muscle were mainly involved in 35 metabolic pathways, and the greater the influence factor of the pathway, the greater the influence on the metabolic pathway (Figure 6A). Differential metabolites were mainly enriched in six metabolic pathways, namely, biotin metabolism; amino acyl-tRNA biosynthesis; lysine degradation; ABC transporters; valine, leucine and isoleucine biosynthesis; and cysteine and methionine metabolism (Table 8).

Differential metabolites in leg muscle were mainly involved in 45 metabolic pathways (Figure 6B). Differential metabolites were mainly enriched in eight metabolic pathways, namely, mTOR signaling pathway; amino acyl-tRNA biosynthesis; *FoxO* signaling pathway; taurine and hypotaurine metabolism; arginine and proline metabolism; cysteine and methionine metabolism; histidine metabolism; and glycine, serine and threonine metabolism (Table 9).

### 3.7. qRT-PCR

#### 3.7.1. Gene Screening

According to the metabolic pathway results of differential metabolites, relevant pathways were identified and genes related to fat deposition were screened out for qRT-PCR (Table 10). Gene selection results are shown in Table 11.

#### 3.7.2. Relative Expression

Relative expression levels of differential genes between CH and Y are shown in Figure 6C. The expression levels of *PPARG*, *FABP3*, *ACSL5*, *FASN*, *UCP3* and *SC5D* in muscles of CH were lower than those of Y, negatively correlated with the fat deposition process. The expression levels of *PPARα*, *ACACA* and *ACOX1* in muscles of CH were higher than those of Y, indicating a positive correlation with the fat deposition process.

## 4. Discussion

Most local chickens have a unique genetic background that makes their meat quality and flavor unique. In this study, CH and Y both contain good local chicken ancestry.

The conventional evaluation indicators of chicken quality mainly include pH, shear force, meat color, dehydration rate, etc. These physical indicators determine the acceptability of meat products. The pH value can reflect the speed of muscle glycogenolysis after poultry slaughter, and it is an important basis for identifying the quality of meat. After the animal is slaughtered, the sugars in the body will be broken down into substances such as lactic acid, which will lower the pH of the muscle mass [24]. In this study, the pH of the breast and leg muscles of Y at 45 min after slaughter was higher than in CH. When the pH was measured after 24 h, it could be seen that the pH of the breast and leg muscles of Y and CH decreased. In addition, Y decreased more. During the 24 h period, the muscles were in the acid excretion stage, which would increase the acidity. The pH change trend of the Y and CH females and males was consistent. Meat color is an important appearance indicator of meat quality, which directly affects consumers’ purchasing desire. The higher the L value of the meat color, the higher the gloss and the paler the color of the meat. The a value is proportional to the meat quality, and the b and L values are inversely proportional to the meat quality [25]. In this study, the L and b values of the breast and leg muscles of Y were higher than those of CH, but the a value of the breast and leg muscles of CH was higher than that of Y. It can be seen that the appearance index of the meat quality of CH is slightly higher than that of Y. The results showed that the meat quality of CH was better. Shear force can be used to evaluate the tenderness, softness, juiciness and other characteristics of cooked meat products, which can intuitively reflect the tenderness of chicken. Shear force was significantly affected by the breed of chicken. In a certain range, the smaller the shear force of chicken, the more tender the meat [26]. In this study, the shear force of the breast and leg muscles of the female and male Y was higher than in CH, so the meat of CH was more tender. The dehydration rate refers to the ability of muscles to retain water. Muscle water contains a lot of amino acids, vitamins, myoglobin, glycogen, mineral ions, etc. The degree of the dehydration rate directly affects the edible quality, including the shape, texture, flavor, texture and juiciness of the meat. The water-holding force of the muscle is generally reflected by the dehydration rate—the greater the dehydration rate, the smaller the water-holding force of the muscle [27]. In this study, the dehydration rate of the breast and leg muscles of the female and male Y was higher than in CH. A high rate of water loss may result in a loss of nutrients contained in muscle water, which can affect the meat quality of the chicken. The conventional chemical components of chicken include water, CF, CP and ash. Determining the content of chemical components is one of the ways to judge the quality of meat. The water in the muscle has a great influence on the tenderness, palatability and juiciness of the meat [28]. CF in the muscle will affect the taste and aroma of the meat. The right amount of fat can ensure the water retention of the meat, so that it has a good taste and tenderness. CP content in muscle is the main source of dry matter difference, and crude ash is the oxidized state of mineral elements in muscle [29]. The chemical composition of muscle determines nutritional value. Typically, muscles with low water content have higher dry matter and protein content and higher nutritional value, but less muscle palatability, juiciness and tenderness. In this study, the contents of water, CF and ash in breast and leg muscles of female and male Y were higher than those of CH to varying degrees. The CP content of the breast and leg muscles of the female and male CH was significantly lower than that of the Y. According to reports, the moisture content of chicken is generally in the range of 70% to 75%. Within the normal range, the higher the moisture content, the better the taste. Therefore, the taste of Y was slightly better than that of CH, but the moisture content of CH was also within the normal range, and the quality of chicken also met the needs of consumption. The higher the fat content within a certain range, the better the quality of the chicken, but if the content is too high, the muscles are easily spoiled. In this study, the fat content of Y was higher than that of CH, so Y had better taste and chicken quality. The ash content, to some extent, represents the mineral content of the chicken. The higher the ash content, the more mineral content or variety in the muscle. Therefore, the mineral content in Y is better than that in CH. As one of the indicators that has attracted much attention in chicken quality research, protein can be hydrolyzed to provide essential amino acids and taste substances needed by the human body [30]. Therefore, CH has an advantage in terms of protein content. In this study, we mainly explored the difference in chicken quality between the two chicken breeds from the physical and chemical indicators of the breast and leg muscles of the female and male Y and CH. From the results, we can conclude that the meat quality of CH was better than that of Y in the measurement results of physical indicators, but the chemical index of Y was better than that of CH. The chemical index results of CH were also within the normal range, and the meat quality of CH can meet the needs of consumers. At present, the evaluation system of meat quality is mainly divided into two aspects: sensory and objective. The slaughter age, gender, rearing environment and diet types are also factors that affect chicken quality. Therefore, the differences in the quality of different breeds of chicken still need to be studied by measuring more comprehensive indicators.

The type, quantity and composition ratio of amino acids constitute the basis for evaluating the level of muscle nutrition, and are also indicators of muscle quality and flavor. Free amino acids in muscles generate aromatic compounds through the Maillard reaction, and are important precursors for taste and flavor [31]. This study showed that the TAA content of CH was higher, and the UAA content was also higher. Among the 17 kinds of AA, the Glu content was the highest; the six kinds of AA with great differences were Asp, Glu, Met, Arg, Ala and Tyr, which included UAA, EAA and NEAA. The composition and content of AA showed that the muscle flavor of CH was better than that of Y.

FA types and contents differences in muscle lead to differences in flavor [32]. The muscle flavor differences mainly come from the fat decomposition and oxidation. Oxide generated by the decomposition of oxidation products continues to generate ketone, aldehyde, acid and olefine aldehyde compounds, also involved in the Maillard reaction. Olefine aldehyde compounds, especially, are an important precursor of muscles to produce aromatic substances. This study showed that under the same feeding conditions, the TFAC of CH was lower than that of Y, but the FA content of C16:0, C18:2N6, C18:3N3, C14:1 and C15:1 in muscle CH was higher than that of Y. This difference may be related to the germplasm characteristics between varieties, indicating that varieties have a great influence on the fatty acid content in muscle.

Metabolomics is the qualitative or quantitative detection and analysis of small molecule metabolites (usually less than 1000 relative molecular weights) in biological fluids, tissues and cells. The number of metabolites is very small compared with the number of genes or proteins, so metabolomics does not need tedious sequencing steps, and can reflect the changes in the functional status of organisms caused by genes or the environment in real time, which is helpful for scholars to understand the metabolism of livestock and poultry at the molecular level [33]. This study showed that differential metabolites in Y and CH breast and leg muscle tissues were identified, with a total of 42 metabolites identified in breast muscle tissue and a total of 55 metabolites in leg muscle tissue. A total of 85 differential metabolites were found in the muscle tissue of the two chickens, mainly divided into eight types: benzene-containing compounds, nucleoside and nucleotide acid analogues, lipids and lipid molecules, organic acids and their derivatives, organic heterocyclic compounds, organic oxygenates, phenylpropane and polyketone, and alkaloids and their derivatives. Among them, the metabolites with the greatest differences in breast muscle were γ-L-glutamyl-L-cysteine, bovinic acid, homo-L-arginine and L-malic acid. The metabolites with the greatest differences in leg muscle were indolin-2-one, (R)-5,6-dihydrothymine and isonicotinic acid. This is consistent with the results of amino acid content and fatty acid content. KEGG pathway enrichment of breast muscle elucidated differential metabolites mainly concentrated in six metabolic pathways: biotin metabolism; amino acyl-tRNA biosynthesis; lysine degradation; ABC transporter; valine, leucine and isoleucine biosynthesis; and cysteine and methionine metabolism. KEGG pathway enrichment of leg muscle elucidated differential metabolites mainly concentrated in eight metabolic pathways: mTOR signaling pathways; amino acyl-tRNA biosynthesis; *FoxO* signaling pathways; taurine and hypotaurine metabolism; arginine and proline metabolism; cysteine and methionine metabolism; histidine metabolism; and glycine, serine and threonine metabolism. Most of the 13 different metabolic pathways were related to amino acid metabolism, which was speculated to be related to the difference in meat quality between CH and Y. The biotin metabolic pathway is one of the important metabolic pathways, and biotin is a necessary factor of long-chain unsaturated fatty acids. It is also related to the synthesis of acetylcholine and the metabolism of cholesterol, which can accelerate the synthesis of fat [34]. A lack of biotin in domestic chickens will increase palmitic acid and linoleic acid, decrease stearic acid and arachidonic acid, and lead to abnormal AA metabolism and lipid metabolism [35]. This study showed that there were certain differences in fat, AA and FA contents and types and structures of CH and Y, consistent with the differences in this metabolic pathway. The biotin contents in CH and Y might be inconsistent due to the different genetic backgrounds, which ultimately lead to differences in meat quality between the two chicken breeds. The amino acyl-tRNA biosynthesis pathway could affect the synthesis of AA. In this study, AA content of CH was much higher than that of Y, which was related to the difference in the amino acyl-tRNA biosynthesis pathway between the two breeds. The content of extracellular AA could determine mTOR activity, and the activation of the pathway could regulate AA metabolism and ribosome synthesis [36]. The disturbance of the mTOR signaling pathway may lead to the disorder of AA metabolism, which was one of the reasons for the difference in AA content. Meanwhile, the abnormality of the mTOR pathway may lead to the disorder of ribosome synthesis and the change in protein synthesis. In this study, the differences in meat quality caused by external factors were excluded, so internal factors were the main factors affecting the differences in meat quality, and the differences in the mTOR signaling pathway may be the main reason for the differences in internal factors of chicken breeds.

*PPARα* is a key regulator of liver fat metabolism [37]. It can bind with FA and their derivatives to regulate the β-oxidation of FA and transform them into acetyl-coenzyme [38], and regulate lipid metabolism throughout the body by controlling the expression of apolipoprotein and lipoprotein lipase [39,40]. In this study, the expression level of PPARα in Y was lower than that in CH, indicating that there was less fat content in muscle tissue of CH, possibly because PPARα promoted the oxidation of fat. *PPARG* is a core member of adipogenesis [41]. At present, no study has found which gene can initiate adipocyte differentiation in the absence of *PPARG* [42]. In this study, the fat and FA content of CH was significantly lower than that of Y, and the expression level of *PPARG* was also lower than that of Y, indicating that *PPARG* may promote the formation of fat and promote the fat deposition in Y more than in CH. *ACACA* catalyzes acetyl-CoA to monoacyl-CoA malonate during FA synthesis. *ACACA* is highly expressed in tissues with strong lipid synthesis [43,44]. In this study, the expression level of *ACACA* in muscle tissue of CH was significantly higher than that of Y, indicating that lipid decomposition was accelerated and deposition was reduced in CH. FABP3 regulates fat metabolism mainly by participating in the uptake, transport and utilization of intracellular long-chain fatty acids. In this study, the *FABP3* expression level of Y was higher than that of CH, and intramuscular fat content of Y was higher than in CH, indicating that *FABP3* promoted fat deposition in Y. *ACSL5* could transform long-chain fatty acids into fatty acyl-coenzymes, thus affecting lipid synthesis and deposition [45,46]. *ACSL5* had high catalytic activity for C16:0, C16:1, C18:1 and C18:2, while promoting the degree of β-oxidation of FA [47,48,49]. In this study, the *ACSL5* expression level of CH was lower than that of Y. The fat content of CH was extremely low, and the FA content was also significantly lower than that of Y, indicating that *ACSL5* promoted the oxidation of fatty acids, reduced triglyceride deposition and accelerated the fat synthesis process. *FASN* is an important regulatory enzyme in the synthesis of fatty acids, located in the significant gene range of fatty acid components such as C10:0, C12:0, C8:0 and C14:0. It can catalyze the conversion of acetyl CoA and monoyl malonate CoA into fatty acids. In this study, the expression level of *FASN* of CH was lower than that of Y, and the fatty acid content of C10:0, C12:0, C18:2 and C18:1 was also significantly lower than that of Y. *SC5D* catalyzes the synthesis of dehydrocholesterol from sterol [50]. In this study, the *SC5D* expression level of CH was lower than that of Y, indicating that the deficiency of *SC5D* in CH resulted in reduced deposition of sterol compounds. *UCP3* was present in brown fat, and the expression of *UCP3* promotes β-oxidation and regulates lipid metabolism. In this study, the fat content and fatty acid content of CH were lower than those of Y, and the *UCP3* expression level was also lower than that of Y. *ACOX1* is the rate-limiting enzyme in the dehydrogenation of the first step of fatty acid β-oxidation. *ACOX1* could change fatty acid metabolism and affect the deposition of triglycerides [51]. High expression of *ACOX1* inhibits lipid droplet formation. The expression level of *ACOX1* of CH was higher than that of Y, indicating that lipid-forming molecules in CH were inhibited by *ACOX1*, fat accumulation was impeded, fatty acid oxidation was accelerated and the accumulation of fatty acids and fat in the body was affected.

## 5. Conclusions

In this study, the meat quality of Y and CH was measured by physical, chemical and nutritional indicators. The comparison and analysis between the two varieties showed that the physical index of CH was better than that of Y, and the chemical index of Y was better than that of CH. However, the chemical index results of CH are also within the normal theoretical value range. Nutritional value indicators in CH are more abundant than in Y. Comprehensive comparison, the meat quality of CH is relatively good. However, there are still many factors affecting the quality of meat. Therefore, more comprehensive index determination is needed to provide a scientific basis for the production, genetic protection, development and utilization of local chicken breeds in Yunnan Province.

Metabolomics analysis showed that CH and Y had 85 different metabolites, and the differential metabolites were mainly classified into eight categories. KEGG pathway enrichment analysis revealed 13 different metabolic pathways. Differential metabolites and metabolic pathways mainly involved the regulation of muscle meat quality and metabolism of amino acids and fatty acids. The screened *PPARG*, *FABP3*, *ACSL5*, *FASN*, *UCP3* and *SC5D* genes were negatively correlated with muscle fat deposition, and PPARα, *ACACA* and *ACOX1* genes were positively correlated with muscle fat deposition. In future molecular breeding, the genes in this study can be used as molecular screening markers and applied to the molecular breeding of chicken quality traits, in order to reveal the mechanisms of fat deposition in poultry at the molecular level.

## Figures and Tables

**Table 1:** Chemical composition of muscle of two chicken breeds.

Items	♂/♀		Water (%)	CF (%)	CP (%)	Ash (%)
Breast muscle	♂	Y	72.65 ± 0.16	1.17 ± 0.02 *	25.00 ± 0.18	1.53 ± 0.05
		CH	72.20 ± 0.24	0.83 ± 0.06	26.67 ± 0.23 **	1.21 ± 0.07
	♀	Y	72.53 ± 0.19	1.39 ± 0.09 *	24.27 ± 0.37	1.89 ± 0.02 *
		CH	71.43 ± 0.25	1.00 ± 0.03	26.68 ± 0.18 **	1.33 ± 0.08
Leg muscle	♂	Y	73.60 ± 0.13	1.47 ± 0.06 **	23.92 ± 0.32	1.77 ± 0.07
		CH	72.86 ± 0.23	1.01 ± 0.13	25.60 ± 0.21 **	1.20 ± 0.06
	♀	Y	72.94 ± 0.17	1.65 ± 0.09 **	23.99 ± 0.06	1.67 ± 0.06 *
		CH	71.97 ± 0.60	1.21 ± 0.07	25.88 ± 0.11 **	1.08 ± 0.09

Note: Comparison of water, CF, CP and ash contents in the same part of CH and Y; ** indicates extremely significant differences (*p* < 0.01); * indicates significant difference (*p* < 0.05).

**Table 2:** Amino acid content in muscles of males.

Items	Breast Muscle		Leg Muscle	
	Y	CH	Y	CH
Asp	6.20 ± 0.70	8.08 ± 1.57 **	6.90 ± 1.31	8.10 ± 1.59 **
Thr	3.47 ± 0.21	4.03 ± 0.75 *	3.03 ± 0.52	3.10 ± 0.68
Ser	1.37 ± 0.03	2.04 ± 0.60 *	1.70 ± 0.38	2.03 ± 0.43 *
Glu	8.42 ± 0.36	14.07 ± 2.45 **	9.07 ± 2.31	14.11 ± 2.70 **
Gly	5.27 ± 0.74	4.11 ± 0.78	5.80 ± 0.66	4.74 ± 1.08
Ala	4.32 ± 0.58	5.03 ± 0.97 *	3.96 ± 0.79	5.21 ± 1.04 *
Cys	0.36 ± 0.21	0.50 ± 0.13 *	0.32 ± 0.15	0.44 ± 0.16 *
Val	4.03 ± 0.52	4.12 ± 0.91	3.77 ± 0.78	4.21 ± 0.89
Met	0.37 ± 0.04	1.00 ± 0.28 **	0.37 ± 0.09	0.62 ± 0.26 **
Ile	3.92 ± 1.35	5.07 ± 0.87 *	4.40 ± 0.81	5.02 ± 0.92 *
Leu	7.27 ± 0.04	8.91 ± 1.30 *	6.56 ± 1.18	7.34 ± 1.35 *
Tyr	1.41 ± 0.45	2.22 ± 1.00 *	1.12 ± 0.42	1.12 ± 0.43
Phe	3.36 ± 0.22	3.37 ± 0.59	3.19 ± 0.41	4.06 ± 0.66 *
His	3.92 ± 2.25	4.13 ± 1.14	3.93 ± 0.73	4.24 ± 0.73
Lys	5.37 ± 0.45	6.29 ± 1.03	5.17 ± 1.27	5.96 ± 1.46
Arg	4.83 ± 0.48	6.19 ± 1.12 **	5.64 ± 0.91	6.60 ± 1.14 *
Pro	3.74 ± 0.32	3.16 ± 0.52	3.99 ± 0.43	3.12 ± 0.71
UAA	19.31 ± 0.99	27.68 ± 1.40 **	20.29 ± 0.57	27.76 ± 1.10 **
EAA	28.39 ± 0.79	31.09 ± 0.53 *	26.89 ± 0.91	30.32 ± 1.20 *
NEAA	15.71 ± 0.21	15.66 ± 0.45	16.68 ± 0.21	15.25 ± 0.40
TAA	66.84 ± 1.98	79.62 ± 2.23 **	68.00 ± 2.03	77.93 ± 2.59 **

Note: Comparison of amino acid content in the same part of CH and Y males; ** indicates very significant difference (*p* < 0.01), * indicates significant difference (*p* < 0.05). UAA, umami amino acid; EAA, essential amino acid; NEAA, nonessential amino acid; TAA, total amino acid.

**Table 3:** Amino acid content in muscles of females.

Items	Breast Muscle		Leg Muscle	
	Y	CH	Y	CH
Asp	5.78 ± 0.71	8.30 ± 0.53 **	6.19 ± 2.17	7.96 ± 1.43 *
Thr	3.17 ± 0.32	3.19 ± 0.35	3.10 ± 0.90	3.10 ± 0.65
Ser	1.24 ± 0.28	1.77 ± 0.33	1.65 ± 0.48	1.81 ± 0.47
Glu	10.19 ± 1.24	13.06 ± 0.90 **	9.75 ± 3.18	13.49 ± 2.43 **
Gly	4.16 ± 0.50	4.06 ± 0.18	4.81 ± 1.00	4.77 ± 1.14
Ala	4.02 ± 0.52	5.15 ± 0.30 *	4.12 ± 1.33	5.06 ± 0.95 *
Cys	0.31 ± 0.16	0.37 ± 0.07	0.26 ± 0.14	0.38 ± 0.06
Val	4.22 ± 0.46	4.30 ± 0.37	3.66 ± 1.31	4.48 ± 0.75 *
Met	0.39 ± 0.15	0.46 ± 0.10 *	0.30 ± 0.10	0.45 ± 0.19 **
Ile	4.59 ± 0.46	4.78 ± 0.37	4.36 ± 1.33	4.46 ± 0.77
Leu	6.72 ± 0.63	6.85 ± 0.51	6.69 ± 1.96	6.73 ± 1.15
Tyr	1.06 ± 0.38	1.55 ± 0.29 *	1.00 ± 0.45	1.40 ± 0.29 *
Phe	3.26 ± 0.30	3.29 ± 0.23	3.14 ± 0.75	3.71 ± 1.00
His	3.52 ± 0.30	3.61 ± 0.50	3.55 ± 2.35	3.92 ± 0.61
Lys	5.04 ± 0.67	6.08 ± 0.54 *	4.94 ± 1.86	5.98 ± 1.27 *
Arg	5.37 ± 0.59	5.79 ± 0.35	5.72 ± 1.45	5.79 ± 1.09
Pro	2.86 ± 0.37	2.85 ± 0.12	3.35 ± 0.65	3.30 ± 0.69
UAA	20.30 ± 0.99	26.88 ± 1.02 **	20.32 ± 0.11	26.88 ± 1.34 **
EAA	28.09 ± 0.96	28.95 ± 1.00	27.61 ± 0.69	28.89 ± 0.77
NEAA	13.03 ± 0.21	13.83 ± 0.46	13.96 ± 0.40	15.19 ± 0.27
TAA	64.89 ± 1.97	74.45 ± 2.11 **	65.51 ± 1.37	75.75 ± 2.00 **

Note: Comparison of amino acid content in the same part of CH and Y females; ** indicates very significant difference (*p* < 0.01), * indicates significant difference (*p* < 0.05). UAA, umami amino acid; EAA, essential amino acid; NEAA, nonessential amino acid; TAA, total amino acid.

**Table 4:** Fatty acid content in muscles of males.

Items	Breast Muscle		Leg Muscle	
	Y	CH	Y	CH
C6:0	1.114 ± 0.134 *	1.006 ± 0.201	1.086 ± 0.163	1.082 ± 0.173
C8:0	1.040 ± 0.114	0.901 ± 0.135	0.996 ± 0.159 **	0.643 ± 0.084
C10:0	0.658 ± 0.092 *	0.591 ± 0.077	0.867 ± 0.113 **	0.645 ± 0.071
C11:0	ND	ND	ND	ND
C12:0	1.044 ± 0.146	1.140 ± 0.016	1.528 ± 0.214	1.564 ± 0.219
C13:0	2.983 ± 0.298 **	2.108 ± 0.422	3.231 ± 0.485 *	2.700 ± 0.459
C14:0	14.105 ± 1.834	16.016 ± 1.202 *	18.524 ± 2.964	19.810 ± 3.010 *
C14:1T	8.703 ± 0.957	7.748 ± 1.007	8.778 ± 1.141 **	5.887 ± 0.765
C14:1	4.908 ± 0.870	7.927 ± 0.246 **	15.771 ± 2.208	18.749 ± 2.062 *
C15:0	3.631 ± 0.436 *	3.169 ± 0.634	4.325 ± 0.649	4.118 ± 0.576
C15:1T	3.351 ± 0.369	3.591 ± 0.646 *	2.135 ± 0.630	3.985 ± 0.371 **
C15:1	3.409 ± 0.477	3.265 ± 0.425	3.553 ± 0.462 **	2.343 ± 0.375
C16:0	720.745 ± 114.904	842.285 ± 81.702 **	684.595 ± 114.603	818.974 ± 89.047 **
C16:1T	6.634 ± 0.663	5.943 ± 1.189	6.711 ± 1.007 **	4.631 ± 0.509
C16:1	12.574 ± 1.635	12.953 ± 1.425	43.014 ± 6.882 *	45.419 ± 4.959
C17:0	6.176 ± 0.679	5.563 ± 0.723	10.393 ± 1.351 *	8.286 ± 1.409
C17:1T	3.979 ± 0.438	4.768 ± 0.064 *	4.767 ± 0.667	5.144 ± 0.823
C17:1	13.492 ± 1.619	13.809 ± 1.243	10.885 ± 1.633	10.996 ± 1.065
C18:0	685.617 ± 75.418 *	636.980 ± 50.958	743.631 ± 118.981 **	530.792 ± 58.387
C18:1N12T	4.529 ± 0.634	4.418 ± 0.574	5.105 ± 0.664 **	3.611 ± 0.506
C18:1N9T	1.867 ± 0.261	1.818 ± 0.031	2.156 ± 0.302 *	1.465 ± 0.249
C18:1N7T	13.713 ± 1.371	13.097 ± 1.572	13.746 ± 2.062 **	8.928 ± 1.429
C18:1N12	103.707 ± 13.482 *	98.733 ± 17.772	202.139 ± 32.342 **	150.504 ± 19.566
C18:1N9C	133.790 ± 14.717	159.243 ± 14.332 *	315.618 ± 48.830	375.589 ± 35.815 **
C18:1N7	32.803 ± 3.608	33.130 ± 3.644	46.322 ± 7.885	54.113 ± 6.176 **
C18:2N6T	2.581 ± 0.310	2.374 ± 0.475	2.704 ± 0.406 *	1.671 ± 0.284
C19:1N12T	7.972 ± 0.877	7.232 ± 0.795	8.779 ± 1.405 **	6.573 ± 1.052
C19:1N9T	4.343 ± 0.608	4.018 ± 0.522	4.480 ± 0.582 **	3.460 ± 0.450
C18:2N6	134.054 ± 18.768	135.419 ± 14.896	302.921 ± 46.609	337.498 ± 33.825 *
C20:0	7.470 ± 0.747	6.620 ± 1.324	10.259 ± 1.539 *	7.660 ± 1.072
C18:3N6	1.544 ± 0.201	2.182 ± 0.393 *	2.949 ± 0.472	3.471 ± 0.590 *
C20:1T	10.219 ± 1.124 *	7.928 ± 1.031	12.548 ± 1.631 *	8.232 ± 1.317
C20:1	6.060 ± 0.667	6.743 ± 0.115	13.620 ± 1.907	17.000 ± 2.210 *
C18:3N3	5.032 ± 0.604	7.294 ± 1.459 *	22.183 ± 3.327	33.338 ± 3.667 **
C21:0	ND	ND	ND	ND
C20:2	10.939 ± 1.203	12.480 ± 1.123 *	15.213 ± 1.476	17.138 ± 1.256 *
C22:0	0.972 ± 0.136	0.989 ± 0.129	1.660 ± 0.346	2.397 ± 0.237 *
C20:3N6	9.218 ± 1.290 *	6.624 ± 0.113	11.758 ± 1.646 **	8.422 ± 1.347
C22:1N9T	5.662 ± 0.566	5.308 ± 1.062	5.703 ± 0.855 **	3.646 ± 0.474
C22:1N9	6.024 ± 0.783	5.642 ± 1.015	6.644 ± 1.063 *	5.240 ± 0.576
C20:3N3	1.688 ± 0.186	1.829 ± 0.238	1.818 ± 0.236 *	1.865 ± 0.191
C20:4N6	157.671 ± 17.344 *	135.847 ± 10.460	254.602 ± 35.644 **	147.414 ± 25.060
C23:0	ND	ND	0.480 ± 0.072 **	ND
C22:2	2.697 ± 0.297	2.600 ± 0.468	2.850 ± 0.456 **	1.672 ± 0.217
C20:5N3	2.707 ± 0.379	2.484 ± 0.323	2.665 ± 0.346 *	2.073 ± 0.228
C24:0	0.580 ± 0.081	0.504 ± 0.086	1.435 ± 0.201 **	0.628 ± 0.088
C24:1	8.811 ± 0.881	7.800 ± 1.092	9.314 ± 1.397 **	6.543 ± 1.112
C22:4	31.969 ± 4.156 *	28.105 ± 3.373	49.593 ± 7.935 **	34.142 ± 5.463
C22:5N6	29.764 ± 3.274 **	11.473 ± 1.491	14.647 ± 1.904 *	12.268 ± 1.595
C22:5N3	31.388 ± 3.453	35.297 ± 3.883	29.285 ± 5.500	39.901 ± 3.289 **
C22:6N3	40.012 ± 4.801 *	33.988 ± 5.098	49.281 ± 7.392 **	23.135 ± 3.239
SFA	1553.587 ± 60.761	1520.852 ± 70.393	1623.739 ± 113.001 **	1265.857 ± 83.291
MUFA	391.129 ± 41.241	402.943 ± 50.201	805.201 ± 62.122 **	663.502 ± 32.174
PUFA	450.325 ± 40.226 *	405.517 ± 40.293	787.256 ± 40.110 **	606.370 ± 54.231
EFA	300.883 ± 15.241 *	283.117 ± 11.321	615.359 ± 22.541 **	493.392 ± 20.231
TFA	2406.952 ± 145.035	2342.781 ± 131.113	3234.067 ± 240.142 **	2552.263 ± 152.216

Note: Comparison of fatty acid content in the same part of CH and Y males; ** indicates very significant difference (*p* < 0.01), * indicates significant difference (*p* < 0.05). SFA, saturated fatty acid; MUFA, monounsaturated fatty acid; PUFA, polyunsaturated fatty acid; EFA, essential fatty acid; TFA, total fatty acids.

**Table 5:** Fatty acid content in muscles of females.

Items	Breast Muscle		Leg Muscle	
	Y	CH	Y	CH
C6:0	1.149 ± 0.115 *	0.928 ± 0.074	0.986 ± 0.140 *	0.908 ± 0.119
C8:0	1.278 ± 0.141 *	0.910 ± 0.087	0.922 ± 0.092	0.914 ± 0.135
C10:0	0.675 ± 0.081	0.687 ± 0.062	0.707 ± 0.078	0.715 ± 0.062
C11:0	ND	ND	ND	ND
C12:0	1.004 ± 0.160	1.290 ± 0.086 *	1.432 ± 0.136	1.651 ± 0.116 *
C13:0	2.697 ± 0.378	2.458 ± 0.270	2.412 ± 0.234	2.044 ± 0.184
C14:0	19.443 ± 2.139 **	12.957 ± 1.814	27.720 ± 3.049 **	15.416 ± 1.449
C14:1T	8.139 ± 0.977	10.346 ± 1.552 **	6.260 ± 0.751	6.309 ± 0.820
C14:1	7.382 ± 1.476	8.864 ± 0.709 *	9.236 ± 1.904	11.397 ± 1.368 **
C15:0	4.221 ± 0.422 *	3.560 ± 0.342	4.453 ± 0.668 *	3.559 ± 0.463
C15:1T	3.339 ± 0.367	5.456 ± 0.600 **	2.350 ± 0.235	2.990 ± 0.350
C15:1	3.384 ± 0.406	4.669 ± 0.434 **	2.493 ± 0.274	2.609 ± 0.261
C16:0	634.506 ± 108.486	857.207 ± 72.293 **	697.576 ± 66.270	748.156 ± 82.297 *
C16:1T	6.165 ± 0.863	6.867 ± 0.961	4.970 ± 0.482	5.093 ± 0.458
C16:1	47.377 ± 5.212 **	15.897 ± 2.384	110.689 ± 12.176 **	28.464 ± 2.676
C17:0	7.700 ± 0.924 *	6.689 ± 0.535	9.657 ± 1.159 **	6.372 ± 0.828
C17:1T	3.919 ± 0.784	4.278 ± 0.353 *	4.407 ± 0.436	4.810 ± 0.457 *
C17:1	12.387 ± 1.239	13.818 ± 1.300	9.114 ± 1.367	11.359 ± 1.477 *
C18:0	641.735 ± 70.591 *	569.937 ± 53.004	558.381 ± 55.438	558.121 ± 78.137
C18:1N12T	4.987 ± 0.598	4.165 ± 0.458	5.094 ± 0.560 *	3.814 ± 0.381
C18:1N9T	2.069 ± 0.269	1.745 ± 0.244	2.194 ± 0.208 *	1.580 ± 0.174
C18:1N7T	12.487 ± 1.748	11.700 ± 1.755	9.736 ± 0.944	10.368 ± 0.933
C18:1N12	230.296 ± 25.333 **	122.932 ± 9.835	172.366 ± 16.760 *	153.055 ± 16.267
C18:1N9C	393.985 ± 47.278 **	190.700 ± 18.307	486.654 ± 58.398 **	247.513 ± 32.177
C18:1N7	49.226 ± 9.845 **	22.913 ± 2.520	75.423 ± 7.467 **	27.762 ± 3.331
C18:2N6T	1.353 ± 0.235	2.146 ± 0.200 **	1.367 ± 0.280	1.923 ± 0.250 *
C19:1N12T	7.524 ± 0.828	6.727 ± 0.740	6.967 ± 0.697	7.164 ± 1.003
C19:1N9T	3.944 ± 0.473	3.548 ± 0.497	3.531 ± 0.388	3.999 ± 0.400
C18:2N6	227.792 ± 29.613 **	106.984 ± 16.048	320.797 ± 30.476 **	189.911 ± 20.890
C20:0	8.267 ± 1.157 *	6.836 ± 0.547	8.607 ± 0.835 *	7.047 ± 0.634
C18:3N6	2.596 ± 0.286 *	1.877 ± 0.180	3.342 ± 0.368	3.183 ± 0.299
C20:1T	10.850 ± 1.302	9.427 ± 1.037	11.147 ± 1.338	10.135 ± 1.318
C20:1	10.870 ± 2.174 **	5.691 ± 0.529	17.465 ± 1.729 **	9.097 ± 1.092
C18:3N3	17.258 ± 1.726 **	5.903 ± 0.649	31.776 ± 4.766 **	14.703 ± 1.911
C21:0	ND	ND	ND	ND
C20:2	9.809 ± 1.079 *	7.362 ± 1.031	13.007 ± 1.301 **	8.615 ± 1.206
C22:0	1.311 ± 0.157	0.985 ± 0.148	1.427 ± 0.157 *	0.921 ± 0.092
C20:3N6	12.975 ± 1.687 **	8.355 ± 0.668	12.286 ± 1.167 *	10.603 ± 1.166
C22:1N9T	4.867 ± 0.681	4.224 ± 0.405	3.908 ± 0.379	3.206 ± 0.379
C22:1N9	5.195 ± 0.571	4.314 ± 0.475	6.151 ± 0.677	5.506 ± 0.518
C20:3N3	1.368 ± 0.164 *	1.007 ± 0.094	1.353 ± 0.162 *	1.007 ± 0.131
C20:4N6	160.657 ± 32.131	137.094 ± 15.080	173.462 ± 17.173 *	128.855 ± 15.463
C23:0	ND	ND	ND	ND
C22:2	2.294 ± 0.252 *	1.863 ± 0.279	1.690 ± 0.169	1.630 ± 0.256
C20:5N3	3.100 ± 0.372 **	1.980 ± 0.158	1.897 ± 0.209	1.755 ± 0.176
C24:0	0.678 ± 0.088	0.454 ± 0.044	0.665 ± 0.063 **	0.344 ± 0.038
C24:1	8.070 ± 1.130	6.706 ± 0.738	6.729 ± 0.653	6.420 ± 0.668
C22:4	35.123 ± 3.864 **	20.616 ± 1.917	35.356 ± 3.889 **	23.653 ± 2.223
C22:5N6	13.578 ± 1.6298 *	7.715 ± 0.849	15.091 ± 1.811 *	13.495 ± 1.754
C22:5N3	22.139 ± 4.428 *	15.839 ± 2.217	24.443 ± 2.420 *	16.273 ± 1.953
C22:6N3	44.336 ± 3.434	39.308 ± 5.896	43.908 ± 6.586 *	37.290 ± 4.848
SFA	1530.839 ± 60.423 *	1275.676 ± 40.341	1313.626 ± 60.102	1349.857 ± 54.154
MUFA	829.206 ± 110.560 **	450.145 ± 107.216	942.727 ± 140.553 **	577.909 ± 100.201
PUFA	535.569 ± 60.403 **	350.686 ± 40.201	667.269 ± 21.005 **	444.482 ± 43.124
EFA	410.655 ± 40.321 **	254.003 ± 20.113	531.245 ± 60.100 **	338.575 ± 45.211
TFA	2906.734 ± 120.021 **	2084.853 ± 114.121	2938.056 ± 159.501 **	2381.784 ± 156.421

Note: Comparison of fatty acid content in the same part of CH and Y females; ** indicates very significant difference (*p* < 0.01), * indicates significant difference (*p* < 0.05). SFA, saturated fatty acid; MUFA, monounsaturated fatty acid; PUFA, polyunsaturated fatty acid; EFA, essential fatty acid; TFA, total fatty acids.

**Table 6:** Basic information of different metabolites identified in breast muscle.

Metabolites	Classification
Vanillylmandelic acid	Benzene-containing compounds
Gallic acid	Benzene-containing compounds
Gentisic acid	Benzene-containing compounds
Adenosine	Nucleoside and nucleotide acid analogues
Nicotinamide ribotide	Nucleoside and nucleotide acid analogues
Lithocholyltaurine	Lipids and lipid molecules
2-Oxo-4-methylthiobutanoic acid	Lipids and lipid molecules
Dethiobiotin	Lipids and lipid molecules
Bovinic acid	Lipids and lipid molecules
γ-L-Glutamyl-L-cysteine	Organic acids and their derivatives
L-Lysine	Organic acids and their derivatives
Homo-L-arginine	Organic acids and their derivatives
12-Hydroxydodecanoic acid	Organic acids and their derivatives
Asymmetric dimethylarginine	Organic acids and their derivatives
1-Aminocyclopropanecarboxylic acid	Organic acids and their derivatives
2-Ketohexanoic acid	Organic acids and their derivatives
1-Methylhistidine	Organic acids and their derivatives
N6-Acetyl-L-lysine	Organic acids and their derivatives
Ergothioneine	Organic acids and their derivatives
L-Malic acid	Organic acids and their derivatives
L-Glutamine	Organic acids and their derivatives
L-Threonine	Organic acids and their derivatives
(R)-3-Hydroxybutyric acid	Organic acids and their derivatives
L-Valine	Organic acids and their derivatives
1-Pyrroline-4-hydroxy-2-carboxylate	Organic heterocyclic compounds
N-Acetylserotonin	Organic heterocyclic compounds
Indolin-2-one	Organic heterocyclic compounds
Glutethimide	Organic heterocyclic compounds
Theophylline	Organic heterocyclic compounds
D-1-Piperideine-2-carboxylic acid	Organic heterocyclic compounds
2-Furoate	Organic heterocyclic compounds
4-(2-Aminophenyl)-2,4-dioxobutanoic acid	Organic oxygenates
Shikimic acid	Organic oxygenates
β-D-Fructose	Organic oxygenates
Biotinyl-5-AMP	Hydrocarbon derivatives
8-Amino-7-oxononanoate	N
3-Methyl-L-tyrosine	N
1-Arachidonoylglycerol	N
Ophthalmate	N
5-Hydroxymethylfurfuryl alcohol	N
2,4-Dinitrophenol	N
3-Methyloxindole	N

**Table 7:** Basic information of different metabolites identified in leg muscle.

Metabolites	Classification
3-Methylindole	Benzene-containing compounds
Creatinine	Benzene-containing compounds
Hexadecanedioate	Benzene-containing compounds
Protoporphyrinogen IX	Nucleoside and nucleotide acid analogues
N6-Acetyl-L-lysine	Nucleoside and nucleotide acid analogues
Prostaglandin F2a	Nucleoside and nucleotide acid analogues
Indolin-2-one	Lipids and lipid molecules
AMP	Lipids and lipid molecules
Rosmarinic acid	Lipids and lipid molecules
5-Methylthioadenosine	Lipids and lipid molecules
Apigenin	Lipids and lipid molecules
Ophthalmate	Lipids and lipid molecules
5-Hydroxyindoleacetic acid	Lipids and lipid molecules
L-Proline	Organic acids and their derivatives
Glucose 6-phosphate	Organic acids and their derivatives
L-Arginine	Organic acids and their derivatives
3-Carbamoyl-2-phenylpropionaldehyde	Organic acids and their derivatives
L-Methionine	Organic acids and their derivatives
O-Acetylcarnitine	Organic acids and their derivatives
4-(2-Aminophenyl)-2,4-dioxobutanoic acid	Organic acids and their derivatives
Creatine	Organic acids and their derivatives
3-Dehydroecdysone	Organic acids and their derivatives
2,4-Dinitrophenol	Organic acids and their derivatives
Phenylethylamine	Organic acids and their derivatives
2-Methylbutanal	Organic acids and their derivatives
Taurocholic acid	Organic acids and their derivatives
Geranyl diphosphate	Organic acids and their derivatives
Xanthine	Organic acids and their derivatives
Anabasine	Organic heterocyclic compounds
Isonicotinic acid	Organic heterocyclic compounds
Hypoxanthine	Organic heterocyclic compounds
Aminocaproic acid	Organic heterocyclic compounds
L-Lactic acid	Organic heterocyclic compounds
Norselegiline	Organic heterocyclic compounds
Antibiotic JI-20A	Organic heterocyclic compounds
Lithocholyltaurine	Organic heterocyclic compounds
dGMP	Organic oxygenates
(R)-3-Hydroxybutyric acid	Organic oxygenates
L-Glutamic acid	Organic oxygenates
β-D-Fructose	Organic oxygenates
3-Methyloxindole	Phenylpropane and polyketone
1-palmitoylglycerophosphocholine	Phenylpropane and polyketone
5,7-Dihydroxyflavone	Phenylpropane and polyketone
Taurine	Alkaloid and their derivatives
5,6-Dihydro-5-fluorouracil	N
2-Ketobutyric acid	N
(R)-5,6-Dihydrothymine	N
γ-L-Glutamyl-L-cysteine	N
N-Acetylhistamine	N
β-Glycerophosphoric acid	N
Hippuric acid	N
S-Lactoylglutathione	N
Ergothioneine	N
L-Threonine	N
(S)-4-Hydroxymandelate	N

**Table 8:** Pathways of KEGG enrichment of different metabolites in breast muscle.

Metabolic Pathway	Total Metabolites	Different Metabolites
Biotin metabolism	28	4
Aminoacyl-tRNA biosynthesis	52	4
Lysine degradation	50	3
ABC transporters	138	5
Valine, leucine and isoleucine biosynthesis	23	2
Cysteine and methionine metabolism	63	3

**Table 9:** Pathways of KEGG enrichment of different metabolites in leg muscle.

Metabolic Pathway	Total Metabolites	Different Metabolites
mTOR signaling pathway	4	2
Aminoacyl-tRNA biosynthesis	52	5
*FoxO* signaling pathway	5	2
Taurine and hypotaurine metabolism	22	3
Arginine and proline metabolism	78	5
Cysteine and methionine metabolism	63	4
Histidine metabolism	47	3
Glycine, serine and threonine metabolism	50	3

**Table 10:** Basic information of related pathways.

Fat Deposition Pathways	Classification	Metabolites
β-Alanine metabolism	Metabolism of other amino acids	L-Aspartic acid; Anserine; Uracil; β-Alanine; L-Histidine; Carnosine; Pantothenate
Histidine metabolism	Amino acid metabolism	Anserine; 3-Methylhistidine; Carnosine; L-Histidine; Ergothioneine; 2-Oxoglutarate
mTOR signaling pathway	Signal transduction	AMP; L-Arginine
Sphingolipid metabolism	Lipid metabolism	Sphingosine 1-phosphate; Sphingosine; L-Serine
*PPAR* signaling pathway	Endocrine system	9-cis-Retinoic acid; 9(S)-HODE; Leukotriene B4
Fatty acid degradation	Lipid metabolism	Glutaryl-CoA; Long-chain fatty acid; Glutarate; 2-Hydroxy fatty acid
Fatty acid biosynthesis	Lipid metabolism	Palmitoleic acid; Hexadecanoate Hexadecanoyl-[acp];
Steroid biosynthesis	Lipid metabolism	Ergocalciferol; Ergosterol; Episterol; Zymosterol
Linoleic acid metabolism	Lipid metabolism	(13S)-Hydroxyoctadecadienoic acid; 13(S)-HPODE; Linoleate
Citrate cycle (TCA cycle)	Carbohydrate metabolism	L-Malate; 2-Oxoglutarate

**Table 11:** Screening gene information.

Gene	Gene Name
*PPARα*	peroxisome proliferator-activated receptor α
*PPARG*	peroxisome proliferator-activated receptor γ
*ACACA*	acetyl-CoA carboxylase α
*FABP3*	fatty acid binding protein 3
*ACSL5*	acyl-CoA synthetase long-chain family member 5
*FASN*	fatty acid synthase
*UCP3*	uncoupling protein 3
*SC5D*	sterol-C5-desaturase
*ACOX1*	acyl-CoA oxidase 1

## Data Availability

The data presented in this study are available on request from the corresponding author. The data are not publicly available due to privacy.

## Supplementary Materials

The following supporting information can be downloaded at: https://www.mdpi.com/article/10.3390/genes13081358/s1. Table S1. Diet composition and nutrition level. Table S2. Detailed screening parameters. Table S3. Primer name, sequence, product size and annealing temperature.
