# Supplementary material for: Physicochemical, Nutritional Properties and Metabolomics Analysis Fat Deposition Mechanism of Chahua Chicken No. 2 and Yao Chicken
Source: Genes (Basel). 2022 Jul 28;13(8):1358. doi: 10.3390/genes13081358 (PMC9407069; doi:10.3390/genes13081358)
Supplement: Supplementary file 1 [file genes-13-01358-s001.zip › genes-1793808-supplementary.pdf]

Table S1. Diet composition and nutrition level

Items（unit）	Period I	Period II
Corn（%）	63.25	60.19
Soybean meal（%）	30.26	25.88
Wheat（%）	0.00	10.00
Fish meal（%）	2.50	0.00
CaCO3（%）	1.11	1.06
CaHPO4（%）	1.50	1.50
Met（%）	0.08	0.07
NaCl（%）	0.30	0.30
Premix（%）	1.00	1.00
ME（MJ.Kg-1）	13.02	12.80
CP（%）	20.00	18.60

Note: Main composition of premix (converted to ration per kg): VA 15000U, VE 62.5mg, VK 3.6mg, VBl 3mg, VB2 9mg, VB6 6mg, Cu l2mg, Zn 75mg, Mn 60mg, I 0.35mg, Se 0.15mg, nicotinamide 60mg, D-pantothenic acid 18mg, folic acid 1.5mg, biotin 0.36mg, VB12 0.03mg, Fe 80mg, choline chloride 600mg, And antibacterial growth promoters, antioxidants.

Table S2. Detailed screening parameters

Screening value	Screening threshold
p-value	p-value ≤ 0.05
VIP	VIP ≥ 1
Fold change	fold_change ≥1.5or≤0.667
one-way ANOVA p-value	p-value ≤ 0.05

Table S3. Primer name, sequence, product size and annealing temperature

Name	Sequence	Product size (bp)	Annealing temperature（℃）
PPARA	F：ACGAATGCCAAGGTCTGAGA<br>R：TGCAAGGATGACTCTGGCTT	169	59
PPARG	F：CTTGGCAGAGATTTCCAGCG<br>R：CTGTTCCTGCAGTGGTGATG	241	57
ACACA	F：ATGGGTGGAAGAGTGCGTAT<br>R：TCCAGTCTCCAGGCATTGAG	157	58
FABP3	F：GAGTACATGAAGGCGTTGGG<br>R：ACCAGCTTGCCTCCATCTAG	224	58
ACSL5	F：ATGGGTGGAAGAGTGCGTAT<br>R：TCCAGTCTCCAGGCATTGAG	157	58
FASN	F：CCTGGAGGTGCTCGATATGT<br>R：ATCAAGGAATCCAGGCCCAA	201	59
UCP3	F：CATCAAGGACACACTGCTGC<br>R：CTTGTAGAGGCCAGCAATGC	217	60
SC5D	F：AGCATGCTGTCCTTCCTCTT<br>R：GAAGGGGTAGACGTGGTAGG	192	59
ACOX1	F：GCACGAGGACTTGAACTTCC<br>R：GGCATGAAGAAGCGATCCTG	237	58

β-actin	F：GTGTGATGGTTGGTATGGGC<br>R：CTCTGTTGGCTTTGGGGTTC	258	58
